# Comparative transcriptomic analysis of Illumina and MGI next-generation sequencing platforms using RUNX3- and ZBTB46-instructed embryonic stem cells

**DOI:** 10.3389/fgene.2023.1275383

**Published:** 2024-01-05

**Authors:** Szilárd Póliska, Chahra Fareh, Adél Lengyel, Loránd Göczi, József Tőzsér, Istvan Szatmari

**Affiliations:** ^1^ Department of Biochemistry and Molecular Biology, Faculty of Medicine, University of Debrecen, Debrecen, Hungary; ^2^ Doctoral School of Molecular Cell and Immune Biology, University of Debrecen, Debrecen, Hungary; ^3^ Department of Human Genetics, Faculty of Medicine, University of Debrecen, Debrecen, Hungary

**Keywords:** RNA-seq, sequencing technology, genomics, embryonic stem cell, RUNX3, ZBTB46

## Abstract

**Introduction:** We have previously observed phenotypic and developmental changes upon the ectopic expression of the RUNX3 or the ZBTB46 transcription factors in mouse embryonic stem cell (ESC) derived progenitors. In this study, we evaluated the gene expression profiles of the RUNX3- and the ZBTB46-instructed murine ESCs with RNA-seq testing two next-generation sequencing technologies.

**Methods:** We compared the DNA nanoball-based DNBSEQ G400 sequencer (MGI) with the bridge-PCR-based NextSeq 500 instrument (Illumina) for RNA sequencing. Moreover, we also compared two types of MGI sequencing reagents (Standard *versus* Hot-massive parallel sequencing (MPS)) with the DNBSEQ G400.

**Results:** We observed that both sequencing platforms showed comparable levels of quality, sequencing uniformity, and gene expression profiles. For example, highly overlapping RUNX3- and ZBTB46-regulated gene lists were obtained from both sequencing datasets. Moreover, we observed that the Standard and the Hot-MPS-derived RUNX3- and ZBTB46-regulated gene lists were also considerably overlapped. This transcriptome analysis also helped us to identify differently expressed genes in the presence of the transgenic RUNX3 or ZBTB46. For example, we found that *Gzmb*, *Gzmd*, *Gzme*, *Gdf6*, and *Ccr7* genes were robustly upregulated upon the forced expression of *Runx3*; on the other hand, *Gpx2*, *Tdpoz4*, and *Arg2* were induced alongside the ectopic expression of *Zbtb46*.

**Discussion:** Similar gene expression profile and greatly overlapping RUNX3- and ZBTB46-regulated gene sets were detected with both DNA sequencing platforms. Our analyses demonstrate that both sequencing technologies are suitable for transcriptome profiling and target gene selection. These findings suggest that DNBSEQ G400 represents a cost-effective alternative sequencing platform for gene expression monitoring. Moreover, this analysis provides a resource for exploration of the RUNX3- and ZBTB46-dependent gene regulatory networks.

## 1 Introduction

Next-generation sequencing (NGS) technology has revolutionized genomic and transcriptomic research. The short-read-based (second generation) sequencing methods still represent the standard approach for genomic and mRNA transcriptomic analyses (Goodwin et al., 2016). Illumina NGS technology is the current leader in the short-read sequencing market. This technology is based on bridge PCR amplification combined with reversed chain termination sequencing (Heather and Chain, 2016). However, there are a few other amplification strategies; for example, the MGI technology is based on rolling circle amplification for DNA nanoball (DNB) generation. These DNB arrays can be used for the combinatorial probe anchor synthesis (cPAS) method, which is a polymerase-based stepwise DNA sequencing approach (Fehlmann et al., 2016; Natarajan et al., 2019). In this report, we compared the DNB-based sequencing by MGI DNBSEQ G400 using the bridge-PCR based method with Illumina NextSeq 500. Moreover, we also evaluated the output of the MGI sequencing platform by monitoring the Standard and the recently introduced Hot-massive parallel sequencing (MPS) chemistries. Of note, numerous recent reports have compared the performance of the MGI with the Illumina sequencers, namely, testing targeted, exome, genome, and metagenome sequencing (Mak et al., 2017; Fang et al., 2018; Patch et al., 2018; Xu et al., 2019; Korostin et al., 2020; Lang et al., 2021; Meslier et al., 2022). Moreover, RNA sequencing including bulk and single-cell approaches were performed for cross-platform analyses (Fehlmann et al., 2016; Zhu et al., 2018; Jeon et al., 2019; Natarajan et al., 2019; Patterson et al., 2019; Senabouth et al., 2020; Wang et al., 2021). It is worth mentioning that most of these comparative mRNA transcriptomic studies focused on the global gene expression profiles and the common parameters of the sequencing data. In this report, we also analyzed the differently expressed gene profiles initiated by ectopic expression of transcription factors through cross-platform comparisons.

RUNX3- and ZBTB46-dependent gene expression profiling were selected for this study because we have previously observed strong phenotypic changes upon the forced expression of these transcription factors in embryonic stem cell (ESC)-derived progenitors. We described that ectopic expression of *Runx3* improved the maturation and immunogenicity of the murine ESC-derived DCs (Takacs et al., 2017). RUNX3 is a critical regulator of several immune cell lineages. In particular, this protein is necessary for the differentiation and maintenance of the cytotoxic CD8^+^ T cells. In addition, Langerhans cells, T helper, natural killer, and B-cell development and function are also modulated by RUNX3 (Boto et al., 2018). We also observed that forced expression of *Zbtb46* interfered with the myeloid blood cell development, and it was associated with enhanced erythroid colony formation at the early stage of the ESC differentiation (Boto et al., 2021). *Zbtb46* is strongly expressed in classical DCs but lacking in monocytes or macrophages (Meredith et al., 2012a; Miller et al., 2012; Satpathy et al., 2012). In addition, ZBTB46 was also detected in group 3 innate lymphoid cells (ILC3s), erythroid progenitors, endothelial cells, and hematopoietic stem cells (Satpathy et al., 2012; Liu et al., 2020; Zhou et al., 2022). It was reported that ZBTB46 negatively impacted the DC activation; moreover, it can interfere with the myeloid blood cell differentiation (Meredith et al., 2012b; Satpathy et al., 2012; Boto et al., 2021). However, the target genes and pathways of this transcription factor are still poorly defined.

To further characterize the genomic impact of ZBTB46 and RUNX3, we employed a genome-scale transcript analysis using Illumina and MGI NGS technologies. We found that DNBSEQ G400 generated transcriptomic data comparable to the results obtained using NextSeq 500. Highly overlapping RUNX3- and ZBTB46-regulated gene sets were identified with both platforms.

## 2 Results

### 2.1 Similar global gene expression profiles with the Illumina and the MGI sequencing platforms

**FIGURE 1:**
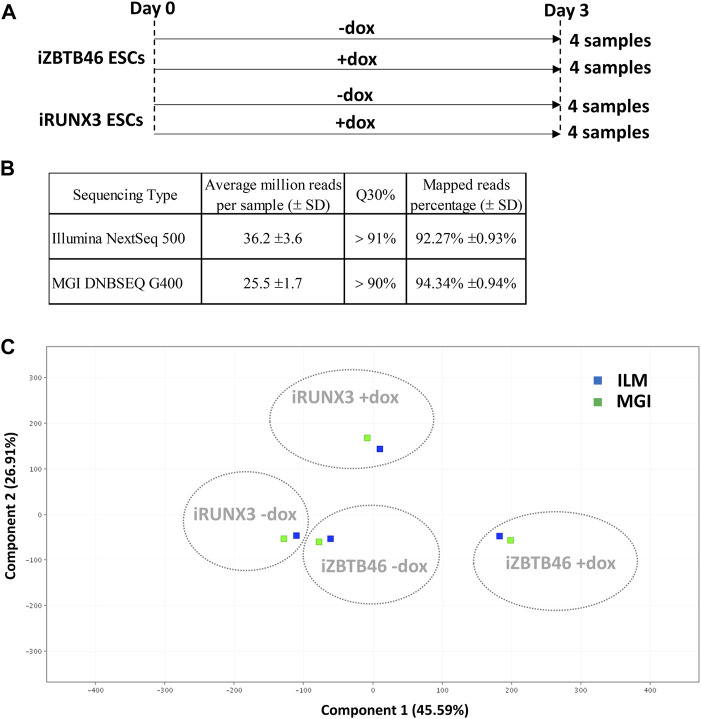
NGS data statistics and global gene expression profiling of the Illumina and the MGI sequencing results. **(A)** Overview of experimental plan: transcriptomic analysis of RUNX3- and ZBTB46-inducible (iRUNX3 and iZBTB46) ESCs. The indicated samples were treated with doxycycline (+dox) for 3 days to induce *Runx3* or *Zbtb46*. Four replicates were used per condition. ESCs were harvested for RNA-seq at day 3. **(B)** Sequencing data statistics. **(C)** PCA for visualizing the platform and transgene-dependent global gene expression changes. For this analysis, 16 Illumina and 16 MGI samples were included, and the replicated samples were combined. The Illumina (ILM)-derived samples were labeled with blue and the MGI-specific samples with green.

In this study, we have compared the Illumina *versus* MGI NGS platforms for transcriptome analysis using RNA samples isolated from transgenic cell lines. For this, doxycycline-inducible mouse ESC clones were applied to monitor the genomic effects of the RUNX3 and the ZBTB46 transcription factors. The chemically inducible ESC lines (iRUNX3 and iZBTB46) were cultured for 3 days in the presence or absence of doxycycline. Four sample sets with four biological replicates were collected; together, 16 samples were applied for RNA sequencing (Figure 1A). The very same RNA samples were sequenced using Illumina NextSeq 500 and in parallel with the MGI DNBSEQ G400 instruments. Both NGS technologies provided good quality of sequencing data with Q30 value >90% and more than 92% of the reads mapped to the reference genome (Figure 1B). Details of library preparation, sequencing, and raw data analyses are described in the Materials and methods section.

First, we evaluated the global gene expression pattern using principle component analysis (PCA) including all transcripts; for this analysis, the four replicates were combined. A distinct gene expression profile was detected upon the ectopic expression of *Runx3* or *Zbtb46*, suggesting that both transgenes elicited robust but disparate changes on the transcript profile (Figure 1C). On the other hand, a similar global gene expression pattern was observed if we compare the Illumina to the MGI RNA sequencing data with PCA (Figure 1C). We also visualized the global expression pattern of the 32 individual samples with PCA (Supplementary Figure S1). In this case, the *Runx3-* and *Zbtb46*-instructed samples still formed distinct populations. Interestingly, the distances among the replicated samples were variable, and in some cases, distances were fairly high; on the other hand, the ILM/MGI-specific distances of the same replicates were modest (Supplementary Figure S1). To further evaluate the gene expression changes, those mRNA transcripts, which are up- or downregulated after *Runx3* or *Zbtb46* inductions, were listed and analyzed.

### 2.2 RUNX3-dependent gene expression signature

**FIGURE 2:**
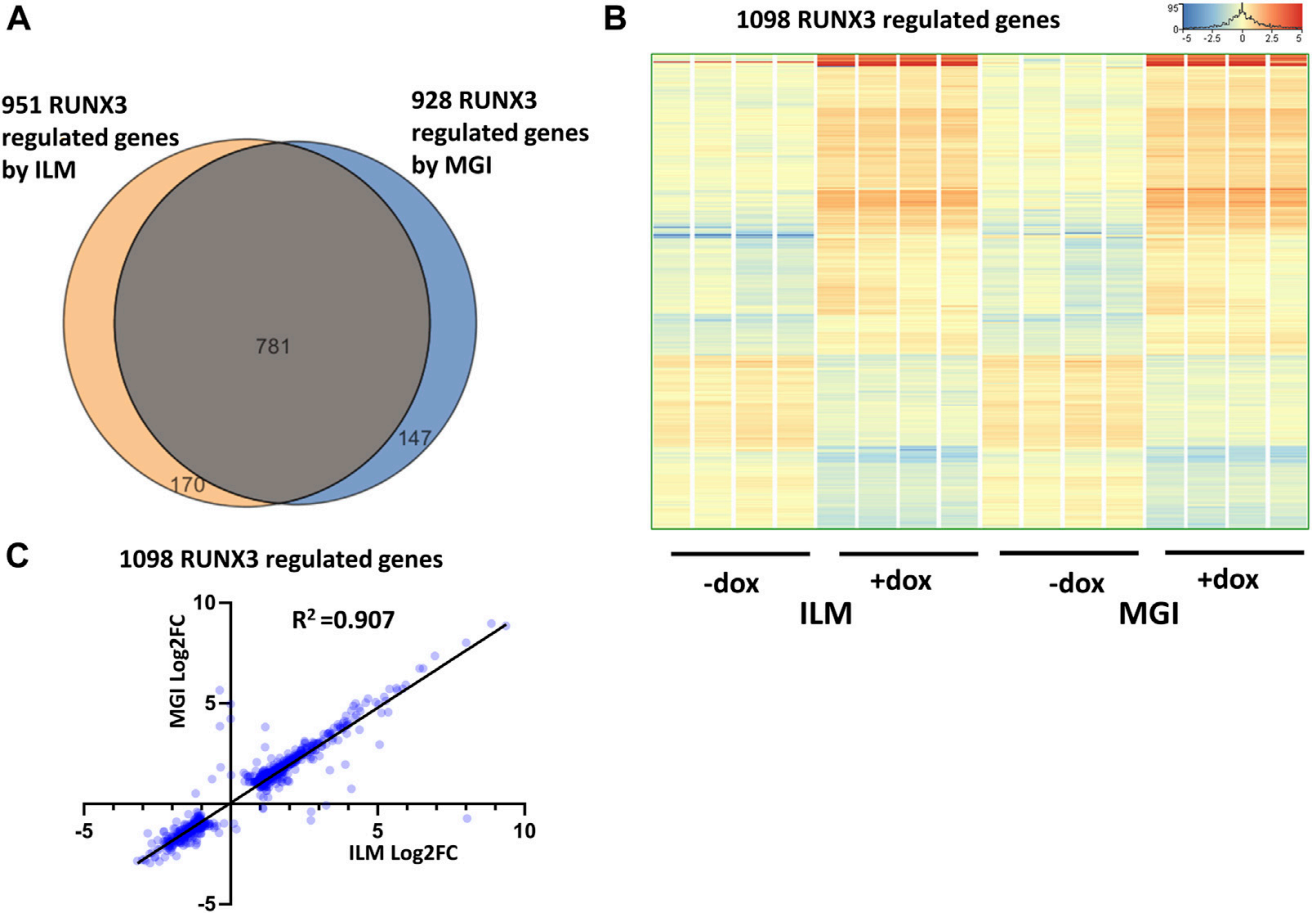
RUNX3-dependent gene expression profile. **(A)** Venn diagram of visualizing overlap of differently expressed genes by RUNX3 between the Illumina (ILM) and the MGI datasets. **(B)** Heatmap showing normalized expression of 1,098 transcripts regulated by RUNX3 after combination of the Illumina and the MGI gene lists. Genes were hierarchically clustered. The indicated samples were treated with doxycycline (+dox) to induce the *Runx3* transgene. **(C)** Scatterplot for pairwise comparison of the Log2-transformed fold change (FC) values calculated from Illumina (ILM) or MGI data. Regression fit is shown in black, with *R* ^2^ value, analyzing 1098 RUNX3-regulated genes.

First, the RUNX3-dependent gene expression changes were investigated by comparing only those samples which were derived from the RUNX3-inducible cells. To select the RUNX3 up- and downregulated genes, the Illumina (eight samples) and MGI (eight samples) sequencing results were separately analyzed. Differently expressed genes were selected with the following criteria: twofold change or greater, *p*-value cutoff was 0.05, and combined with multiple test correction (details are described in the Materials and methods section). In case of Illumina platform 951, RUNX3-regulated transcripts were detected (602 were upregulated, and 349 were downregulated); on the other hand, 928 RUNX3-dependent genes were obtained (590 were upregulated, and 338 were downregulated) from MGI data. The Illumina and the MGI gene lists were profoundly overlapped: 781 genes were found in both lists (Figure 2A). After combining the MGI and Illumina datasets, a hierarchical cluster analysis was employed. Most of the regulated genes in this combined gene list (1,098 entities) showed a similar gene expression pattern in both platforms (Figure 2B). Next, we used a pairwise comparison of the ILM *versus* MGI fold change (FC) values analyzing the 1,098 genes using a scatterplot (Figure 2C). As expected, most of the FC values were similar, although the calculated coefficient of determination (*R* ^2^) was 0.907, because a few genes had a rather distinctive FC. Together, these findings demonstrate that most of the RUNX3-regulated transcripts exhibit a similar expression profile in both platforms. Next, we performed a functional analysis of the regulated genes for identifying overrepresented Gene Ontology (GO) categories testing the RUNX3-regulated combined gene list. Notably, 95 “biological process” categories were overrepresented (Supplementary Table S1). Many of them are related to development and morphogenesis. For example, the “blood vessel development” category, which contains 75 transcripts, was highly overrepresented.

**FIGURE 3:**
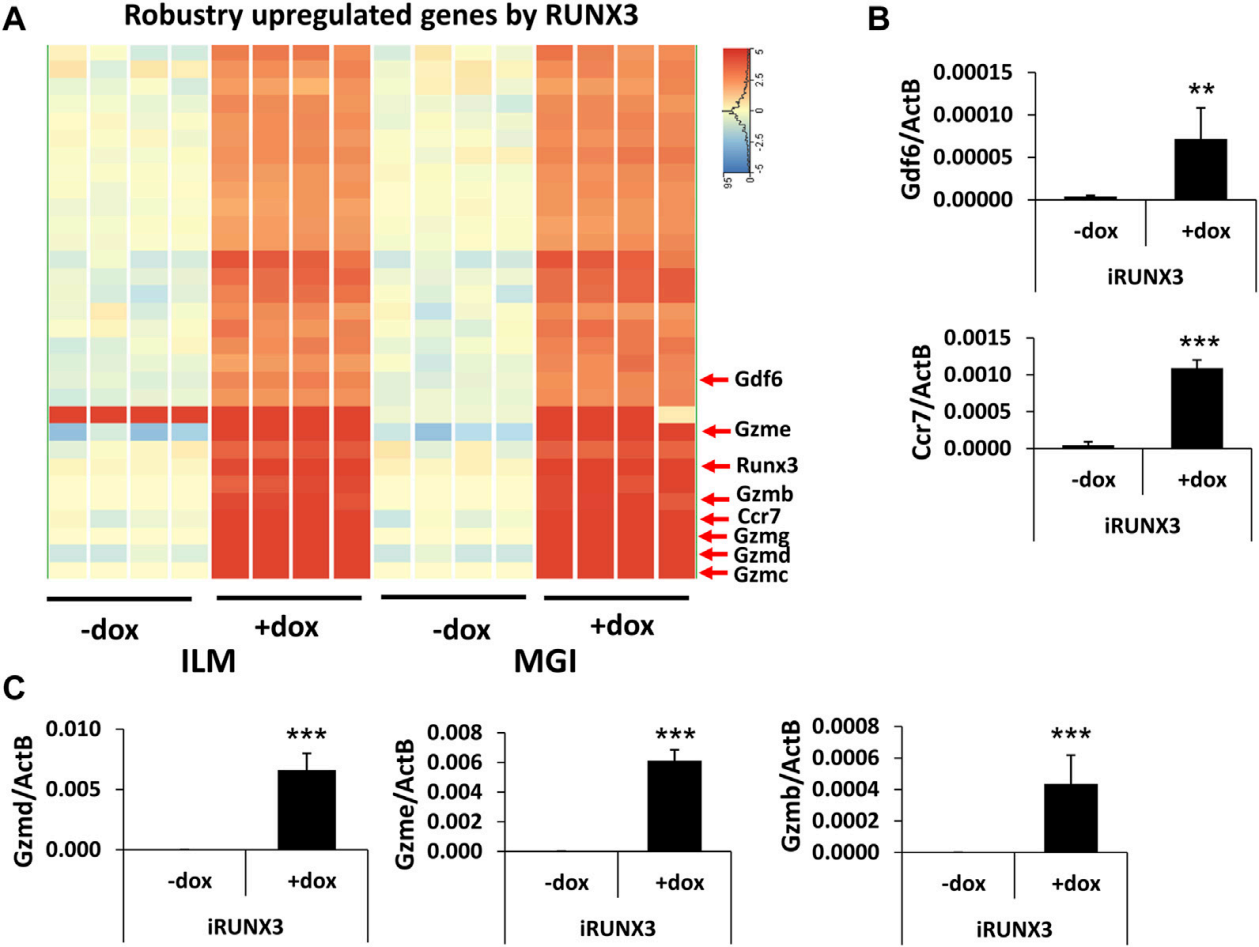
Robustly upregulated genes by RUNX3. **(A)** Hierarchical clustering of the strongly upregulated genes by RUNX3 obtained from the Illumina (ILM) or the MGI datasets. **(B)** Transcript levels of *Gdf6* and *Ccr7* were validated with quantitative RT-PCR using independent RNA samples. **(C)** Transcript levels of *Gzmb*, *Gzmd*, and *Gzme* were validated with quantitative RT-PCR using independent RNA samples. The indicated samples were treated with doxycycline (+dox) to induce *Runx3*. Average and SD values were calculated from six independent experiments. Statistically significant differences compared to the control group (-dox) are presented, with **: *p* < 0.005; ***: *p* < 0.001.

After the general analysis of the RUNX3-regulated genes, we investigated those upregulated genes which exhibited very robust induction (Figure 3A). For example, *Ccr7* and *Gdf6* were greatly upregulated; in addition, several members of the murine granzyme B gene family (*Gzmb*, *Gzmc*, *Gzmd*, *Gzme*, and *Gzmg*) were induced upon the ectopic expression of *Runx3*. We have validated *Ccr7*, *Gdf6*, *Gzmb*, *Gzmd*, and *Gzme* induction using quantitative RT-PCR analysis, testing independent samples (Figures 3B,C). In summary, our genome-scale transcript analysis provides evidence for the specific gene-regulatory role of RUNX3 in undifferentiated ESCs.

### 2.3 ZBTB46-dependent gene expression signature

**FIGURE 4:**
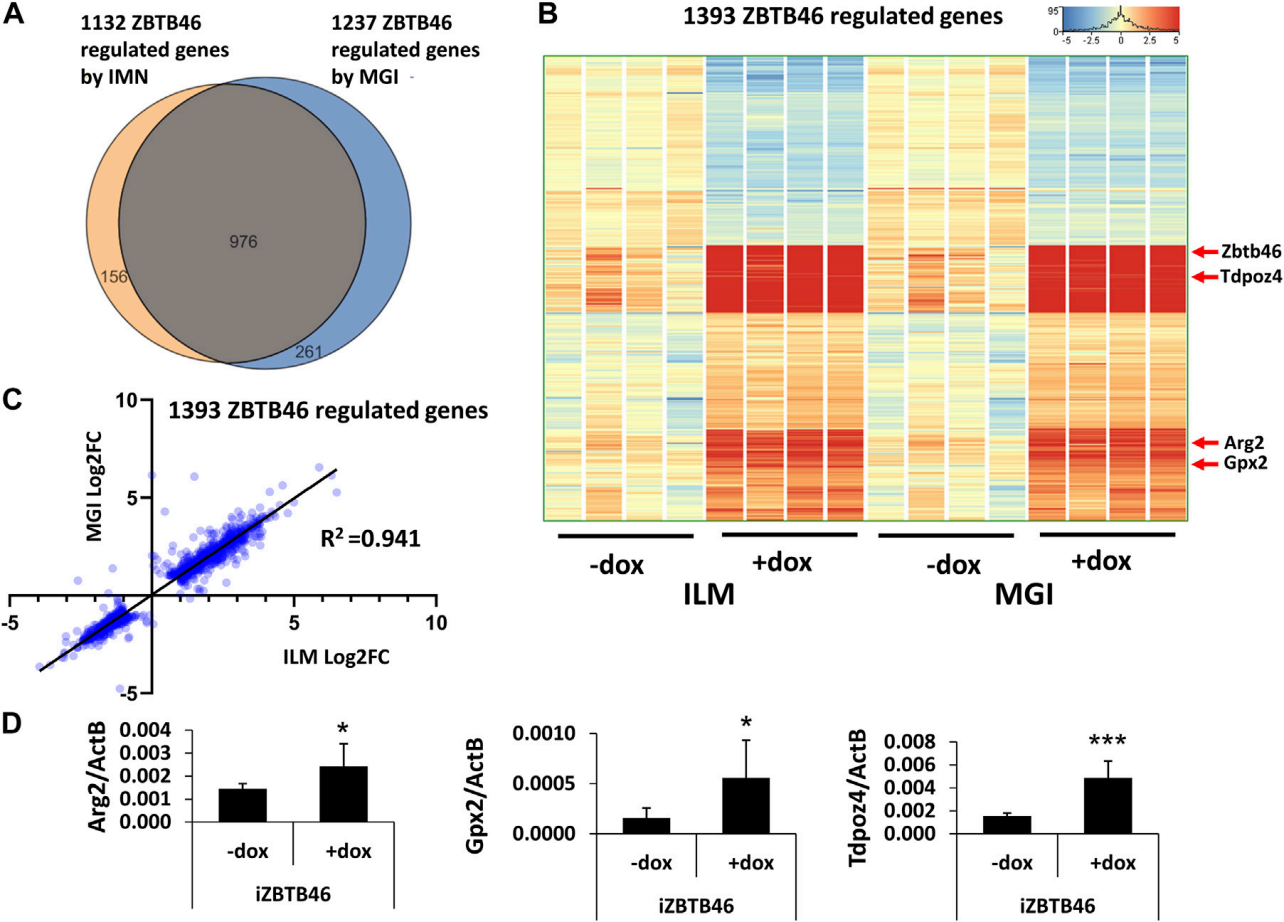
ZBTB46-dependent gene expression profile. **(A)** Venn diagram of visualizing overlap of differently expressed genes by ZBTB46 between the Illumina (ILM) and the MGI datasets. **(B)** Heatmap showing normalized expression of 1,393 transcripts regulated by ZBTB46 after combination of the Illumina (ILM) and the MGI gene lists. Genes were hierarchically clustered. **(C)** Pairwise scatterplot for comparing the Log2-transformed fold change (FC) values calculated from Illumina (ILM) or MGI data. Regression fit is shown in black, with *R* ^2^ value, analyzing 1393 ZBTB46 regulated genes. **(D)** Transcript levels of *Arg2*, *Gpx2*, and *Tdpoz4* were validated with quantitative RT-PCR using independent RNA samples. The indicated samples were treated with doxycycline (+dox) to induce *Zbtb46*. Average and SD values were calculated from six independent experiments. Statistically significant differences compared to the control group (-dox) are presented, with *: *p* < 0.05; ***: *p* < 0.001.

**FIGURE 5:**
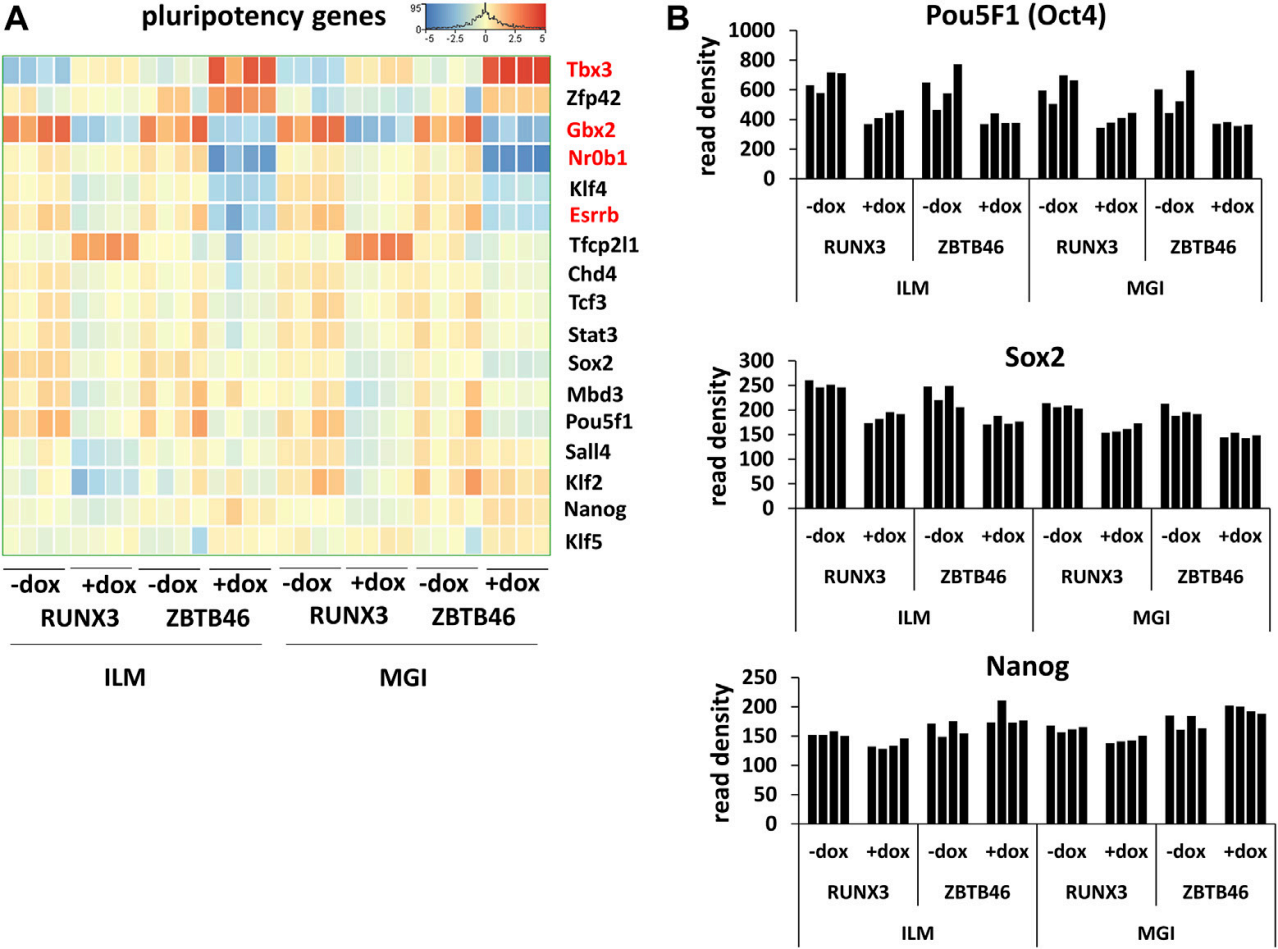
Transcript profile of the pluripotency genes. **(A)** Hierarchical clustering of 17 mouse pluripotency-related genes. Differently expressed genes upon ZBTB46 induction are labeled in red. **(B)** Read densities of *Pou5f1*, *Sox2*, and *Nanog*. Expression results were obtained from the Illumina (ILM) or the MGI datasets. Samples were derived from RUNX3- or ZBTB46-inducible cells. The indicated samples were treated with doxycycline (+dox) to induce *Runx3* or *Zbtb46*.

Next, the ZBTB46-dependent gene expression profile was monitored. To identify the ZBTB46-regulated genes, the same gene selection pipeline was applied as for the RUNX3-inducible cells. Remarkably, 1132 ZBTB46-regulated genes were detected (645 were upregulated, and 487 were downregulated) with the Illumina platform; on the other hand, 1237 ZBTB46-dependent genes were obtained (736 were upregulated, and 501 were downregulated) from the MGI sequencing data. Once again, the two gene lists are firmly overlapped: 976 commonly regulated transcripts were found (Figure 4A). Moreover, the hierarchical cluster analysis of the merged gene list (1,393 entities) indicates almost identical gene expression profiles comparing the two sequencing platforms (Figure 4B). During this analysis, we also recognized sample-specific gene expression patterns. For example, numerous ZBTB46-regulated genes showed a higher baseline expression in case of the second replicate of the non-induced cells (-dox). Remarkably, this trend was observed in both sequencing platforms (Figure 4B). We also implemented a pairwise comparison of the ILM/MGI FC values testing the ZBTB46-regulated gene list (Figure 4C). In this case, *R* ^2^ was 0.941, indicating that the FC values were more similar comparing with the RUNX3-regulated gene list. Together, these findings demonstrate that a large number of ZBTB46-modulated genes were detected, and they exhibited a comparable gene expression profile in both platforms. We managed to validate the enhanced expression of *Tdpoz4*, *Arg2*, and *Gpx2* in the presence of the transgenic ZBTB46 with quantitative RT-PCR testing independent RNA samples (Figure 4D). Next, a functional analysis was performed to study the overrepresented GO categories in the ZBTB46-regulated gene list. Interestingly, multiple cell differentiation- and proliferation-related categories were overrepresented (Supplementary Table S2). Numerous cell differentiation-related GO categories were overrepresented in the ZBTB46-instructed cells; therefore, it is possible that these cells escaped the pluripotent state. To investigate this possibility, we monitored the expression profile of those genes which participate in the regulation and maintenance of the mouse ESC pluripotency (Dunn et al., 2014). Interestingly, four (*Essrb*, *Nr0b1*, *Gbx2*, and *Tbx3*) out of the 17 pluripotency genes exhibited altered expression in the presence of the transgenic ZBTB46 (Figure 5A). On the other hand, only the *Gbx2* gene showed altered expression upon *Runx3* induction. However, when we analyzed the raw expression data (read densities) of the core pluripotency genes (*Pou5f1*, *Sox2*, and *Nanog*), we found that these transcripts were highly expressed in all samples (Figure 5B). However, the *Pou5f1* (also known as *Oct4*) and *Sox2* expressions showed slightly lower read densities in the presence of RUNX3 or ZBTB46. We conclude that the ZBTB46- or the RUNX3-programmed cells are only partially differentiated because the three core pluripotency genes were still highly expressed in these cells. This analysis also revealed that a very similar expression pattern of the individual samples was obtained from the Illumina and the MGI sequencing data if raw data (read density values) were considered.

It is worth mentioning that we have recently performed a global gene expression profiling using ZBTB46-instructed ESC-derived mesodermal cells (Boto et al., 2021). Therefore, we compared the ZBTB46 upregulated and downregulated gene lists derived from the current experiment and the previously published study. Our Venn diagram analysis showed that there was only a moderate overlap among these lists (Supplementary Figure S2), suggesting that different genes are regulated in mesodermal derived cells *versus* undifferentiated ESCs upon the ectopic expression of *Zbtb46*.

### 2.4 Sequencing platform-dependent alterations

**FIGURE 6:**
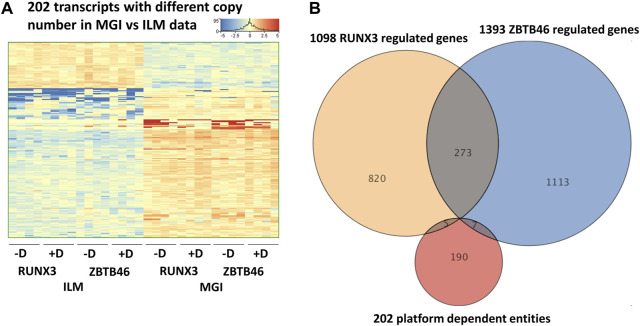
Platform-specific alterations. **(A)** Heatmap showing 202 transcripts with significantly different copy numbers comparing the MGI with the Illumina (ILM) sequencing results. Genes were hierarchically clustered. Samples were derived from the RUNX3- or the ZBTB46-inducible cells. The indicated samples were treated with doxycycline (+D) to induce *Runx3* or *Zbtb46*. **(B)** Venn diagram visualizing overlap of differently expressed genes by RUNX3, ZBTB46, and the platform-dependent genes.

**FIGURE 7:**
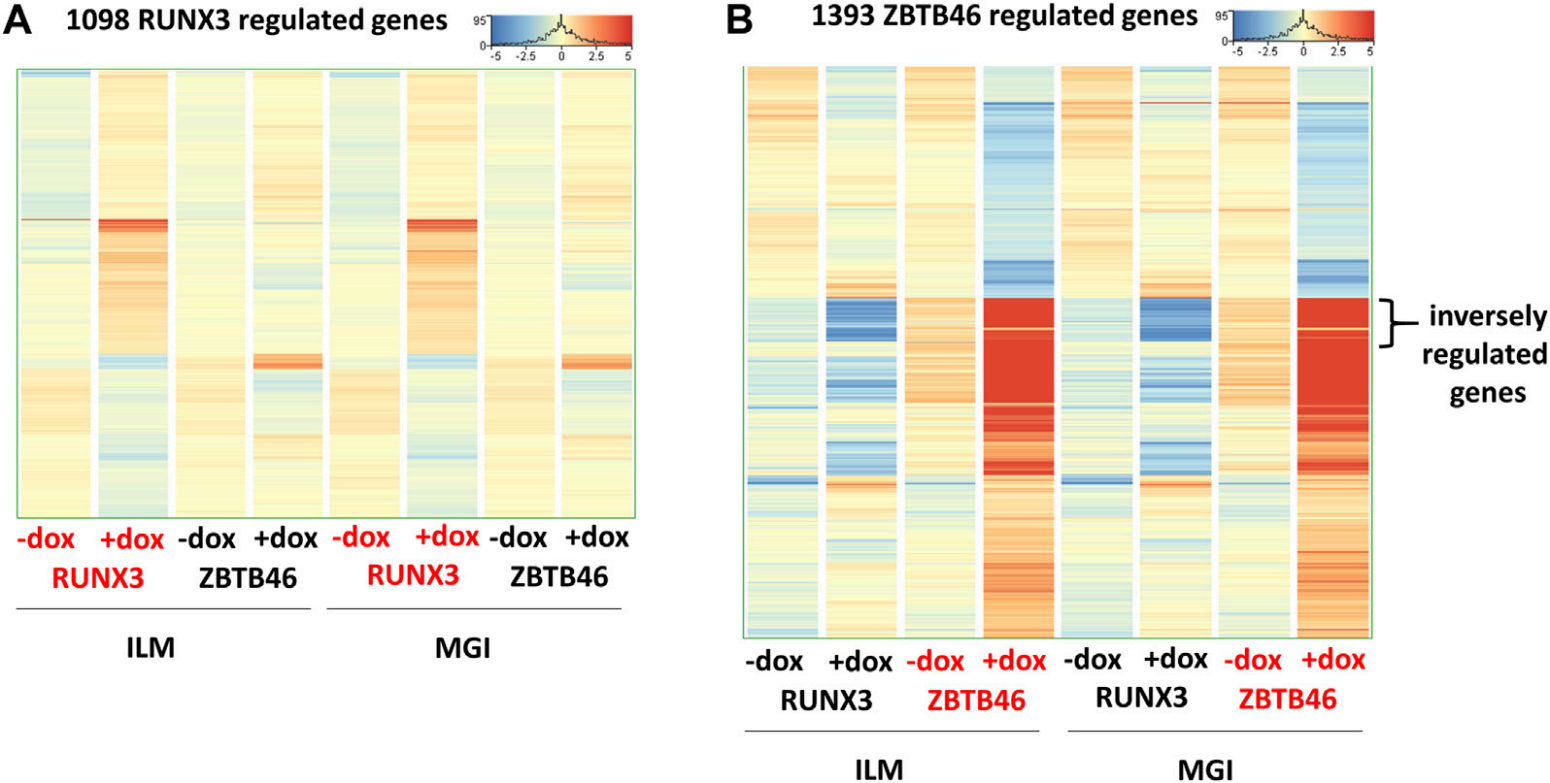
Comparative gene expression profiles of the RUNX3- and the ZBTB46-regulated genes. Heatmaps include both the RUNX3 and the ZBTB46 data obtained from the Illumina (ILM) or the MGI datasets. In these heatmaps, the average expression of the four replicates is shown. Genes were hierarchically clustered. The indicated samples were treated with doxycycline (+dox) to induce *Runx3* or *Zbtb46*. **(A)** Heatmap showing normalized expression of 1,098 transcripts regulated by RUNX3. **(B)** Heatmap showing normalized expression of 1,393 transcripts regulated by ZBTB46. The indicated sector was oppositely modulated.

In this report, we focused on how to examine the ZBTB46- and RUNX3-dependent gene expression changes; however, it is still possible that some transcripts are varied due to the different sequencing technologies independent of the transgene induction. Therefore, we analyzed all expressing genes and selected those transcripts which exhibited platform-specific alterations. For this, we applied the same selection criteria as for the RUNX3- or the ZBTB46-regulated genes. Interestingly, if we compare all of Illumina to all of the MGI samples, 202 out of the 16,052 expressed genes showed differences (Figure 6A). Approximately 75% of the platform-dependent transcripts (154 entities) showed higher copy number in the MGI data. Our cluster analysis also indicated that most of these genes exhibited a rather random expression pattern, suggesting that these changes represent a non-specific fluctuation. In addition, we found that most of these transcripts were pseudogenes with relatively low read density (data not shown). Moreover, there was a minimal overlap between the RUNX3- and ZBTB46-regulated gene lists *versus* the platform-dependent genes (Figure 6B). Together, these findings suggest that only a limited number of transcripts with low copy number exhibit platform-specific changes. This comparative analysis also revealed that 273 overlapping genes were found if we compare the RUNX3 *versus* ZBTB46-regulated genes (Figure 6B). Therefore, we combined the RUNX3- *versus* the ZBTB46-specific samples to identify the putative co-regulated genes (Figure 7). Unexpectedly, we failed to detect many co-regulated genes; instead, numerous ZBTB46-induced genes exhibited a diminished expression upon the forced expression of *Runx3* (Figure 7B), suggesting that multiple genes are oppositely modulated by these factors.

To further characterize the platform-dependent alterations, we carried out pairwise comparisons combining two related RNA samples testing only with Illumina or MGI or in combination of the two sequencing platforms. For this comparative study, the 16,052 expressed genes were used and two sample pairs were selected: replica 1 (R1) and 2 (R2) of the iRUNX3-dox, and R1/R2 of the iZBTB46-dox (Supplementary Figure S3). Based on our PCA results (Supplementary Figure S1), we found that the global expression profile looked similar in case of the two iRUNX3-dox samples; in contrast, R1 *versus* R2 of iZBTB46-dox looked rather distinct. Consistent with this observation, we obtained better similarities (*R* ^2^ = 0.967–0.982) with the iRUNX3-dox samples; in contrast, lower *R* ^2^ values (0.915–0.937) were detected with the iZBTB46-dox replicates (Supplementary Figure S3). However, if we compared the ILM *versus* MGI sequencing data on the very same sample, the coefficient of determination values were fairly high and similar (*R* ^2^ = 0.974–0.978). These results suggest that the platform-dependent alterations are uniform and comparable; in contrast, the sample-dependent transcriptomic changes can fluctuate.

### 2.5 MGI sequencing with Hot-MPS chemistry

**FIGURE 8:**
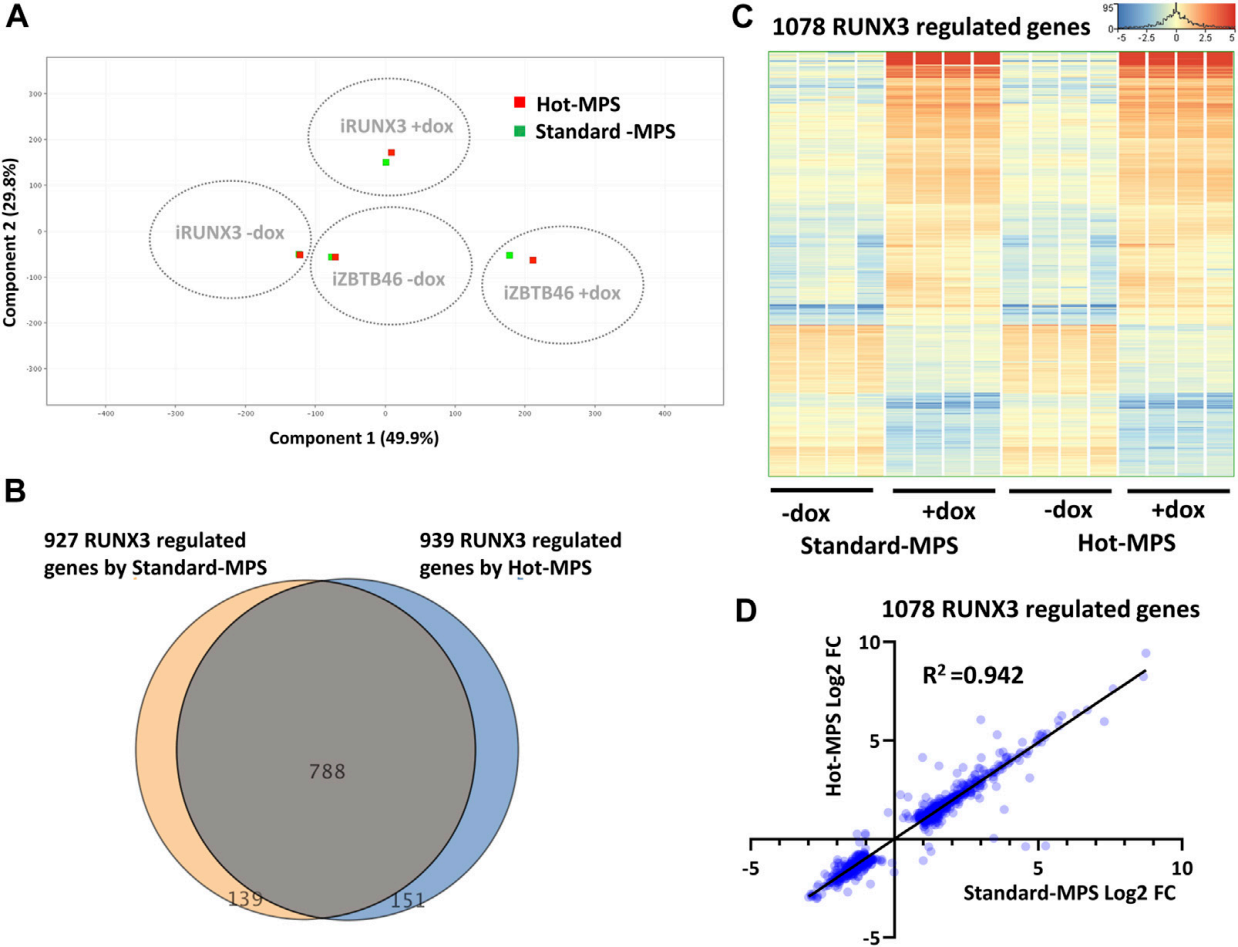
Standard *versus* Hot-MPS MGI sequencing results. **(A)** PCA for visualizing the global gene expression changes. For this analysis, 16 Standard and 16 Hot-MPS samples were included, and the four replicates were combined. The Hot-MPS samples were labeled with red and the Standard-MPS samples with green. The indicated samples were treated with doxycycline (+dox) to induce *Runx3* or *Zbtb46*. **(B)** Venn diagram of visualizing overlap of differently expressed genes by RUNX3 comparing the Standard *versus* Hot-MPS data. **(C)** Heatmap showing normalized expression of 1,078 transcripts regulated by RUNX3 after combination of the two gene lists. Genes were hierarchically clustered. The indicated samples were treated with doxycycline (+dox) to induce *Runx3*. **(D)** Scatterplot for pairwise comparison of the Log2-transformed fold change (FC) values calculated from Standard- and Hot-MPS MGI data. Regression fit is shown in black, with *R* ^2^ value, analyzing 1078 RUNX3-regulated genes.

In this report, we also carried out a comparative study for testing two types of MGI sequencing chemistries. For the inter-platform comparison, the Standard-MPS (Standard-massive parallel sequencing) MGI reagent was used. However, a novel, Hot-MPS chemistry has been recently introduced by MGI. To apply the Hot-MPS chemistry, the DNBSEQ G400 sequencing instrument was updated; thereafter, the ZBTB46- and the RUNX3-inducible samples were re-sequenced with the new chemistry. We used the very same cDNA libraries that were tested with the Standard-MPS sequencing. Importantly, very good quality of sequencing data was obtained with the Hot-MPS reagent: Q30 value >95% and more than 94% of the reads mapped to the reference genome. To compare the Standard *versus* Hot-MPS results, we assessed the global gene expression pattern with PCA, including all MGI samples and RNA transcripts. As expected, distinct expression patterns were associated with the ectopic expression of *Runx3* or *Zbtb46* (Figure 8A). On the other hand, similar global gene expression profiles were detected if we compare the Standard *versus* the Hot-MPS sequencing data. To further evaluate the MGI NGS data, the RUNX3- and ZBTB46-regulated genes were selected testing the Standard and the Hot-MPS sequencing results separately. We used a similar selection as it was applied for the Illumina *versus* MGI comparison. In total, 939 RUNX3-dependent genes were detected (606 were upregulated, and 333 were downregulated), testing the Hot-MPS data. We also observed that the Standard and the Hot-MPS-derived gene lists were considerably overlapped (788 common genes; Figure 8B). Moreover, clustering of the combined gene list (1,078 entities) revealed that most of the RUNX3-regulated genes showed an apparently similar gene expression pattern in both sample sets (Figure 8C). Next, we compared the FC values calculated from the Standard and Hot-MPS data using the combined gene list (Figure 8D). Interestingly, better correlation (*R* ^2^ = 0.942) was detected than the ILM/MGI FC scatterplot (*R* ^2^ = 0.907; Figure 2C). These findings suggest that a similar expression pattern can be extracted from the Hot-MPS sequencing data. Of note, in case of the Standard-MPS analysis, 927 RUNX3-dependent transcripts were found (Figure 8B); this number is slightly different from the RUNX3-regulated transcripts using the MGI data, as shown in Figure 2A. The reason for this alteration is that in this case, normalization was performed only with the MGI sequencing data; therefore, the fold change values could be slightly different.

**FIGURE 9:**
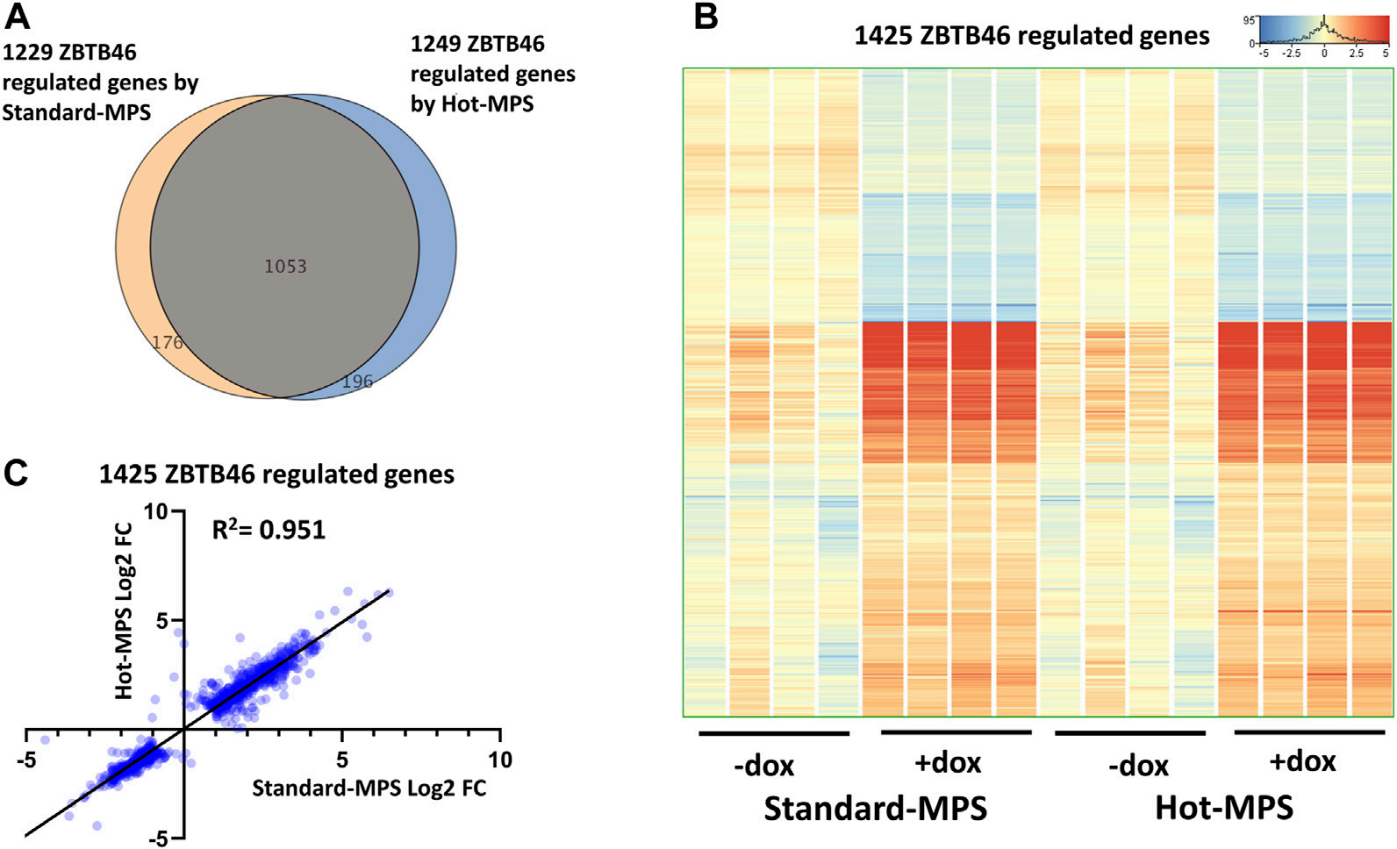
ZBTB46-regulated gene selection using the Standard- and Hot-MPS MGI sequencing results. **(A)** Venn diagram of visualizing overlap of differently expressed genes by ZBTB46 comparing the Standard *versus* Hot-MPS data. **(B)** Heatmap showing normalized expression of 1,425 transcripts regulated by ZBTB46 after combination of the two gene lists. Genes were hierarchically clustered. The indicated samples were treated with doxycycline (+dox) to induce the *Zbtb46* transgene. **(C)** Pairwise scatterplot for comparing the Log2-transformed fold change (FC) values calculated from Standard- and Hot-MPS MGI data. Regression fit is shown in black, with *R* ^2^ value, analyzing 1425 ZBTB46-regulated genes.

**FIGURE 10:**
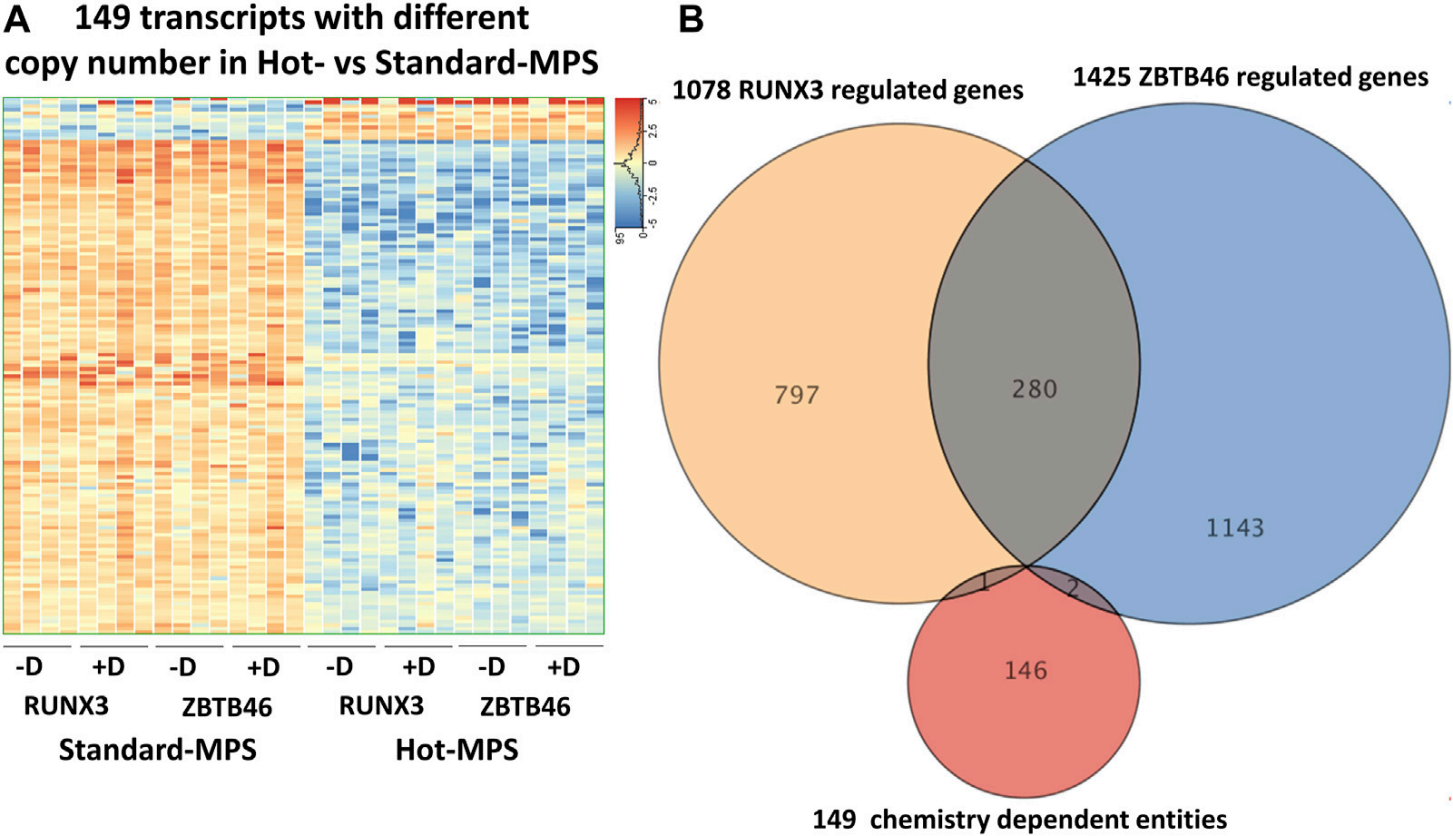
MGI sequencing reagent-specific alterations. **(A)** Heatmap showing expression pattern of 149 transcripts which exhibited significantly different copy numbers comparing the Hot- to the Standard-MPS sequencing results. Genes were hierarchically clustered. Samples were derived from the RUNX3- or the ZBTB46-inducible cells. The indicated samples were treated with doxycycline (+D) to induce *Runx3* or *Zbtb46*. **(B)** Venn diagram visualizing overlap of differently expressed genes by RUNX3, ZBTB46, and the chemistry-dependent gene entities.

Next, the ZBTB46-dependent gene expression changes were defined: 1,249 regulated genes were detected (748 were upregulated and 501 were downregulated by ZBTB46) from the MGI Hot-MPS sequencing data. Once again, the Standard and the Hot-MPS lists were profoundly overlapped: 1,053 commonly regulated transcripts were detected (Figure 9A). Moreover, the hierarchical cluster analysis of the merged gene list (1,425 entities) indicates a visibly similar gene expression pattern comparing the two sequencing chemistries (Figure 9B). In addition, the Standard *versus* Hot-MPS-specific FC values were well correlated (*R* ^2^ = 0.951) upon testing the combined ZBTB46-regulated gene list (Figure 9C). Finally, we selected those transcripts which are changed due to different MGI sequencing chemistries testing all expressing genes. Interestingly, if we compare the Standard to the Hot-MPS samples, 149 out of the 16,190 expressed genes showed alteration. Unexpectedly, most of these gene products exhibited higher copy number in the MGI Standard-MPS dataset (Figure 10A). However, our cluster analysis indicated that many of these genes exhibit a random expression profile; moreover, we found that most of these transcripts were pseudogenes with low read densities. It is important to mention that only three overlapped entities were found between the RUNX3- and ZBTB46-regulated gene lists *versus* the chemistry-dependent genes (Figure 10B). We concluded that typically similar gene expression profiles can be acquired if we compare the Standard *versus* the Hot-MPS sequencing results; only a limited number of transcripts, with low copy number, exhibited MPS chemistry-specific changes.

## 3 Discussion

In this report, we provide a cross-platform comparison of two short-read DNA sequencing technologies. We aimed to analyze the similarities and differences in gene expression using poly-A selected RNA sequencing data generated with the Illumina NextSeq 500 and the MGI DNBSEQ G400 platforms. For this comparative analysis, we tested the same RNA samples derived from RUNX3- or ZBTB46-instructed ESCs. For this NGS study, the single-end read mode was used with 75 sequencing cycles with the Illumina instrument and 100 cycles with the MGI sequencer. For a more accurate comparison, the sequencing read length was adjusted to 75 for the MGI data. Due to the relatively short read length, we have not used mRNA splice variant assessment or SNP analysis; instead, we focused on gene expression profiling. These two prominent NGS platforms have been already compared in numerous studies testing transcriptomic data (Fehlmann et al., 2016; Zhu et al., 2018; Jeon et al., 2019; Natarajan et al., 2019; Patterson et al., 2019; Senabouth et al., 2020; Wang et al., 2021). Consistent with the previous findings, we observed that both sequencing platforms showed comparable levels of quality, sequencing uniformity, and gene expression profiles. For example, our PCA indicated a similar global gene expression pattern comparing the two platforms. In addition, highly overlapping RUNX3- and ZBTB46-regulated gene lists were obtained from both sequencing datasets. Moreover, the cluster analysis of the merged gene list indicates a visibly similar gene expression pattern comparing the two platforms. We also applied pairwise comparisons of the Illumina *versus* MGI FC values testing the RUNX3- or ZBTB46-regulated genes; these analyses further support the cross-platform similarity. These findings suggest that both NGS technologies are suitable for global transcript profiling and target gene selection. In this study, we also compared the Standard *versus* Hot-MPS MGI sequencing reagents with the DNBSEQ G400 instrument. The Hot-MPS sequencing chemistry is also based on a polymerase-dependent stepwise DNA sequencing approach (cPAS-based sequencing) (Fehlmann et al., 2016). For this analysis, the MGI cDNA libraries, which had been used for the cross-platform comparison, were re-sequenced with the Hot-MPS reagent. As expected, we obtained a similar global gene expression pattern comparing the two MGI chemistries; moreover, highly overlapping RUNX3- and ZBTB46-regulated gene lists were obtained from both datasets. We conclude that the Hot-MPS-based MGI sequencing is also a suitable approach for global transcript profiling.

Despite similar gene expression profiles and the overlapping transcription factor regulated gene lists, nevertheless, we also detected platform-dependent gene expression changes. A notable difference between the two sequencing platforms is that the MGI uses linear DNA amplification (rolling circle DNA synthesis); in contrast, the Illumina technology uses PCR amplification to make sequencing arrays (Fehlmann et al., 2016; Heather and Chain, 2016). It is possible that different amplification strategies contribute to the altered copy number of some cDNAs. However, when we compared the two MGI-based chemistries, comparable numbers of alterations were detected (202 *versus* 149 genes). In both cases, most of the entities with an altered transcript level were pseudogenes with relatively low read density. It is also worth mentioning that there was a minimal overlap between the RUNX3- and ZBTB46-regulated gene lists *versus* the platform/reagent-dependent transcripts. Therefore, we concluded that these platform/reagent-specific alterations represent a background fluctuation which was more or less randomly generated and exerted a minimal impact on the transcription factor-mediated gene expression changes.

In parallel with the cross-platform comparison, we also analyzed the genomic impact of the RUNX3 and ZBTB46 on the global gene expression profile of ESCs. The transcriptional landscape of the murine ESCs has been intensively studied using bulk or single-cell mRNA sequencing (Lienert et al., 2011; Kumar et al., 2014; Kolodziejczyk et al., 2015; Malik et al., 2022). Interestingly, it was observed that the gene expression pattern of the murine ESCs is heterogeneous and stochastic; moreover, it is depending on the cell culture conditions (Kumar et al., 2014; Torres-Padilla and Chambers, 2014). In our study, ESCs were cultivated in leukemia inhibitory factor (LIF) plus serum containing medium without feeder cells. It is worth mentioning that using this culture condition, ESCs exhibit heterogeneous expression of the pluripotency factors (Toyooka et al., 2008; Silva et al., 2009). Despite this heterogeneity, our analysis indicated that the core pluripotency genes (*Pou5f1*, *Sox2*, and *Nanog*) were strongly expressed in these cells. On the other hand, some additional pluripotency-related genes (*Essrb*, *Nr0b1*, *Gbx2*, and *Tbx3*) showed an altered expression, especially in the presence of the transgenic ZBTB46. In addition, we also observed that numerous cell differentiation- and development-related GO categories were overrepresented in the ZBTB46- or RUNX3-instructed cells. Therefore, it is possible that enforced expression of the *Zbtb46* or *Runx3* can initiate ESC differentiation. However, it is very likely that only a subset of the cells started to differentiate in the presence of these transgenes because the core pluripotency genes remained expressed in the RUNX3- or ZBTB46-instructed cell populations. In the future, time course experiments and single-cell transcriptome analysis are needed to further characterize the genomic effects of these transcription factors in ESCs.

It is worth mentioning that a huge number of ectopically expressed transcription factors have been already tested in undifferentiated ESCs. Together, 185 transcription factors were induced in mouse ESCs using doxycycline-regulated transgenic cells (Nishiyama et al., 2009; Yamamizu et al., 2013; Yamamizu et al., 2016). Some of these transcription factors elicited robust global gene expression changes based on microarray analyses. Notably, in these previous studies, neither *Runx3* nor *Zbtb46* was probed. Here, we described that the ectopic expressions of the *Runx3* or the *Zbtb46* can also profoundly modify the gene regulatory networks of ESCs. In the future, we intend to use a more detailed analysis and validation of the ZBTB46- or RUNX3-regulated genes and pathways. However, in this report, we have already confirmed that *Gpx2*, *Tdpoz4*, and *Arg2* were robustly upregulated upon the forced expression of *Zbtb46* in ESCs. Moreover, we also confirmed with RT-PCR that *Ccr7* and *Gdf6* were positively regulated by RUNX3. In addition, we found that several members of the granzyme B gene family (*Gzmb*, *Gzmc*, *Gzmd*, *Gzme*, and *Gzmg*) were induced upon the ectopic expression of *Runx3*. Interestingly, *Gzmb* has been already connected with a RUNX3-dependent regulation. Granzymes are highly expressed in cytotoxic T lymphocytes and natural killer cells, and they are involved in immune-targeted cell death (Boivin et al., 2009). RUNX3 has a prominent role during the differentiation of the cytotoxic lymphocytes; moreover, RUNX3 stimulated the expression of the *Gzmb* in cytotoxic T cells (Cruz-Guilloty et al., 2009). Our current analysis suggests a broader regulatory effect of RUNX3 on the granzyme B genes because many members of this gene family were positively regulated. Further genomic and epigenomic studies are needed to characterize the RUNX3-dependent regulation of the granzyme genes.

Collectively, a similar gene expression profile and greatly overlapping RUNX3- and ZBTB46-regulated gene sets were detected with both DNA sequencing platforms. Moreover, an almost identical gene expression pattern was obtained with the MGI Hot-MPS reagent compared to the Standard-MPS chemistry. Therefore, we can conclude that the DNBSEQ G400 system represents a cost-effective alternative platform for gene expression profiling and transcriptome analysis. Moreover, this global gene expression analysis provides a resource for the exploration of the RUNX3- and ZBTB46-dependent gene expression regulation.

## 4 Materials and methods

### 4.1 ESC culture and transgene induction

For this study, RUNX3- and ZBTB46-inducible murine ESC clones (iRUNX3 clone number 4 and iZBTB46 clone number 4) were used (Takacs et al., 2017; Boto et al., 2021). ESCs were cultured without the mouse embryonic fibroblast (MEF) layer in knockout DMEM (Thermo-Fisher Scientific) supplemented with 15% fetal bovine serum (FBS, Thermo-Fisher Scientific), 1,000 U/mL LIF (Merck), 100 μg/mL streptomycin, and 100 U/mL penicillin (Merck). Cell culture medium was refreshed at day 1; cells were harvested with 0.25% trypsin-EDTA (Thermo-Fisher Scientific) after 3 days. Transgenes (*Runx3* or *Zbtb46*) were induced for 3 days by doxycycline treatment (1 μg/mL).

### 4.2 Library preparation and RNA sequencing

Total RNA was extracted using the TRI reagent (Molecular Research Center, Inc., United States), and its integrity was assessed using Agilent BioAnalyzer. Sixteen RNA samples were isolated from the 3-day cultivated RUNX3- and ZBTB46-inducible ESCs (with or without doxycycline treatment; four replicates per condition). RNA sequencing was performed with MGI DNBSEQ G400 and, in parallel, with the NextSeq 500 instrument (Illumina). For the Illumina platform, RNA-seq libraries were prepared from total RNA using the Ultra II RNA Sample Prep kit (New England BioLabs) according to the manufacturer’s protocol. Sequencing runs were executed using single-end 75 cycles sequencing using the NextSeq500/550 High Output Kit v2.5 (Illumina) sequencing reagent. For the MGI platform, RNA-seq libraries were prepared from total RNA using MGIEasy RNA Library Prep Set v3.0 (MGI) according to the manufacturer’s protocol. Single-end 100 cycles sequencing runs were executed on the MGI DNBSEQ G400 instrument using DNBSEQ-G400RS High-throughput Rapid Sequencing Set (FCS SE100; Standard-MPS). The sequencing of the same libraries was repeated in a paired-end 100 cycles sequencing run with Hot-MPS High-throughput Sequencing Set (G400 HM FCL PE100). Library preparation, sequencing, and bioinformatics were accomplished by the Medical Genomics and Bioinformatics Core Facility at the University of Debrecen.

### 4.3 RNA-seq data analysis

The sequencing read length was adjusted to 75 for all of the MGI data, and in case of the Hot-MPS paired-end data, only the read 1 fastq files were used for downstream analysis. Sequenced reads were aligned to the mm10 genome assembly (GRCm38), HISAT2 alignment program was used, and bam files were generated. Further analyses were executed with Strand NGS 4.0 software (Agilent Technologies). Data were normalized using the DEseq algorithm. The normalized counts were log-transformed and baselined to the median of all samples. To select the transgene-regulated transcripts, eight RUNX3-inducible (with or without doxycycline treatment; four replicates per condition) or eight ZBTB46-inducible samples were separately analyzed. To select the MGI *versus* the Illumina or the Standard *versus* the Hot-MPS-specific transcripts, the corresponding 16 samples were compared. First, transcripts were filtered on gene expression setting 1 read density (normalized reads per kilobase of transcript per million mapped reads) cutoff level, and those entities for which at least 2 out of 8 (or 16) samples had higher read density than the cutoff were selected. Thereafter, differently expressed genes were selected with the following criteria: twofold change or greater, and *p*-value cutoff was 0.05 using moderate *t*-test with Benjamini–Hochberg multiple test correction. For hierarchically clustering, similarity was measured by Squared Euclidean of the indicated gene lists. The coefficient of determination (*R* ^2^) values for pairwise comparison of FC or normalized counts were calculated with linear regressions using GraphPad Prism 10.1. In case of the linear regression analyses of the 16,052 expressed genes (Supplementary Figure S3), the normalized counts were not baselined.

### 4.4 Quantitative RT-PCR

Total RNA was prepared using the TRI reagent. Reverse transcription was carried out using the High-Capacity cDNA RT Kit (Thermo-Fisher Scientific). Real-time PCR was performed with an LC96 PCR instrument (Roche). PCR conditions were as follows: one cycle for denaturation at 95°C for 60 s; 40 cycles at 95°C for 10 s, and 60°C for 30 s using TaqMan hydrolysis probe-based assays (Thermo-Fisher Scientific). Assay IDs are listed in Supplementary Table S3. Relative gene expressions were determined using the comparative quantification cycle (Cq) method and normalized to ActB. PCR reactions were carried out in triplicates plus one control without reverse transcriptase enzyme. Relative expression was represented as mean ± SD. Significant differences between mean values were evaluated using a two-tailed Student’s t-test.

## Data Availability

The datasets presented in this study can be found in online repositories. The names of the repository/repositories and accession number(s) can be found online at: https://www.ncbi.nlm.nih.gov/sra/PRJNA926944.
