# Supplementary figures and images for: Comparative transcriptomic analysis of Illumina and MGI next-generation sequencing platforms using RUNX3- and ZBTB46-instructed embryonic stem cells
Source: Front Genet. 2024 Jan 5;14:1275383. doi: 10.3389/fgene.2023.1275383 (PMC10796612; doi:10.3389/fgene.2023.1275383)

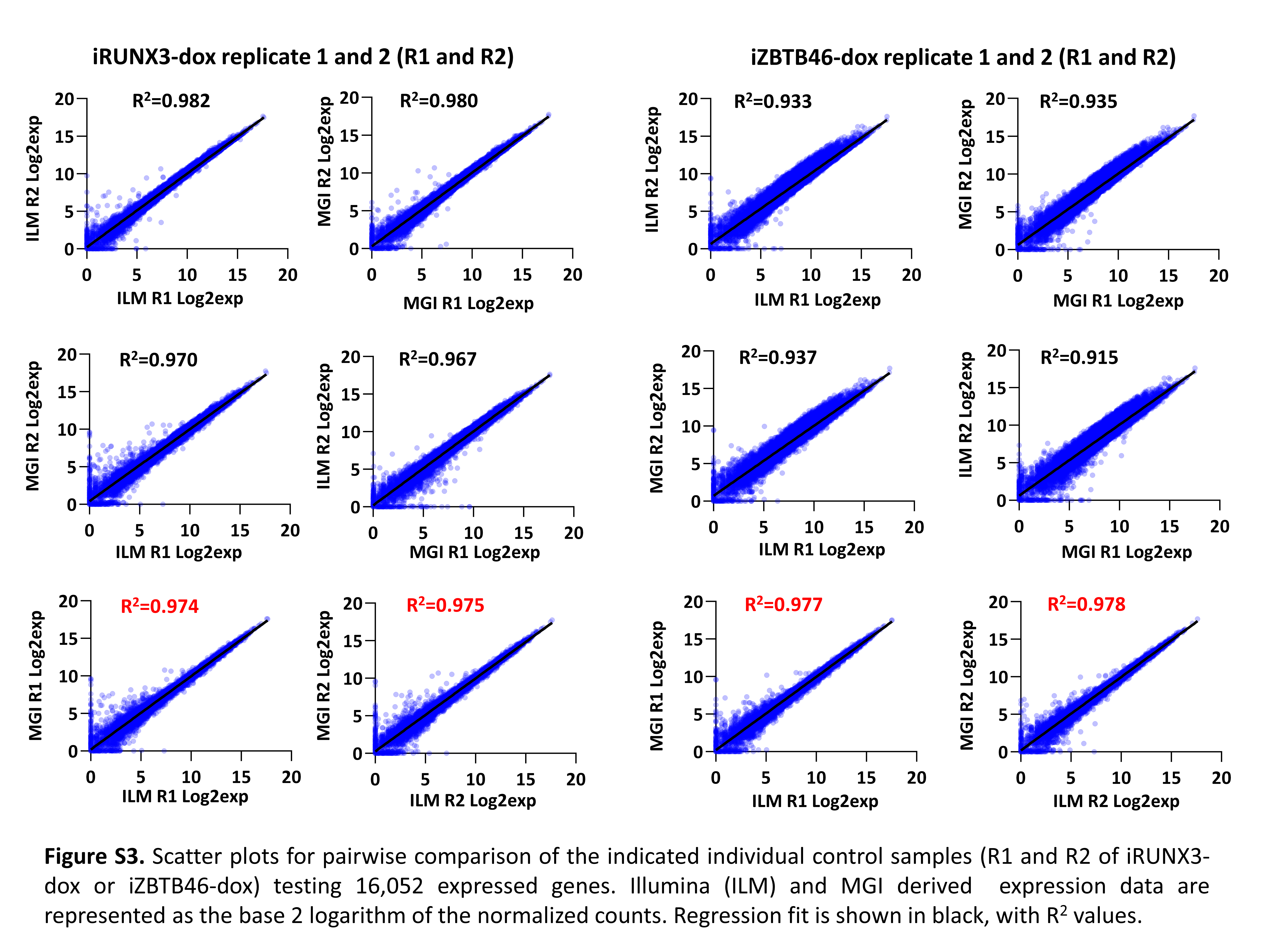

Supplement: Supplementary file 3 [file Image3.TIF]

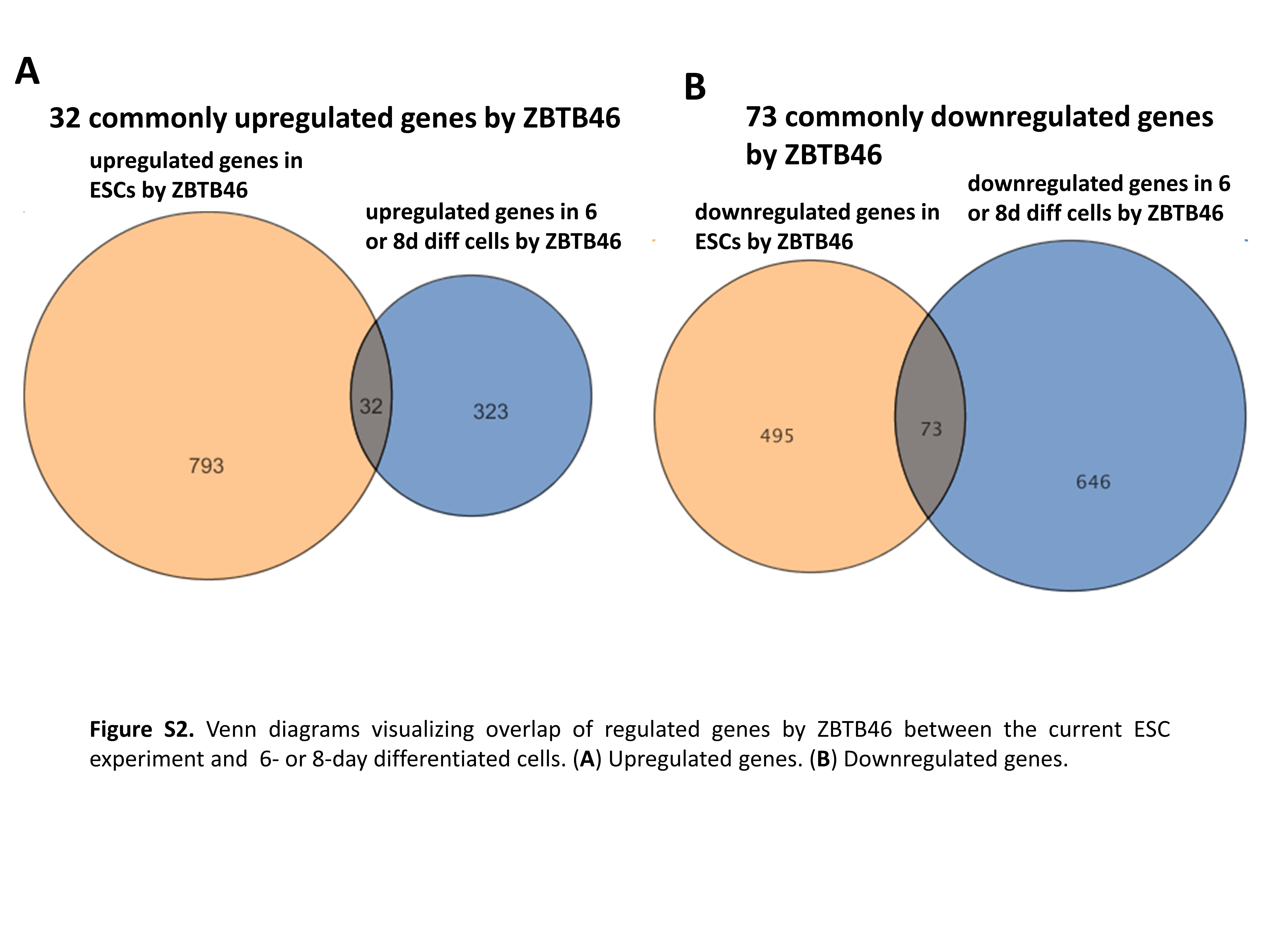

Supplement: Supplementary file 4 [file Image2.TIF]

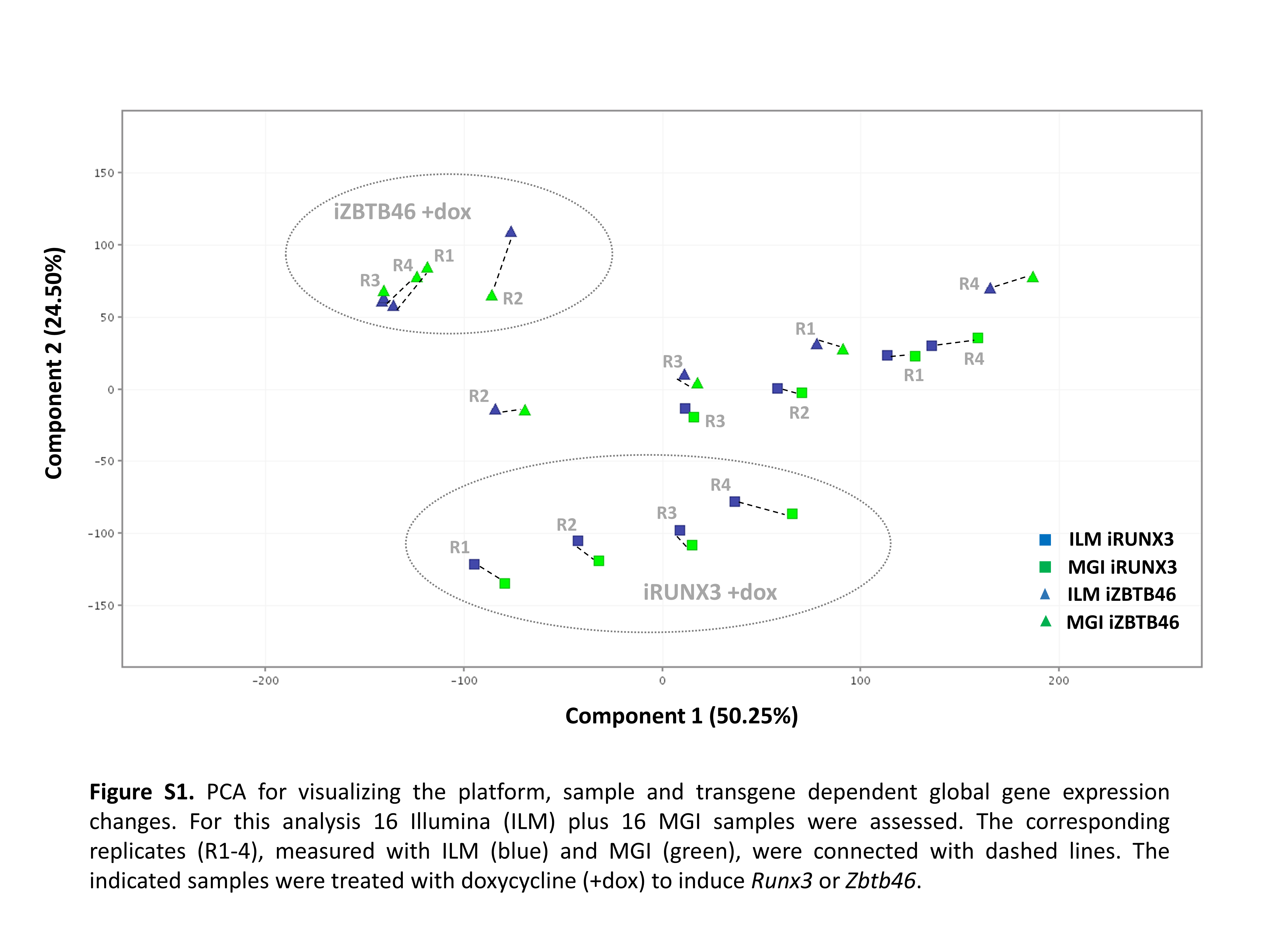

Supplement: Supplementary file 5 [file Image1.TIF]
